# Potential of Cell-Free Supernatant from *Lactobacillus plantarum* NIBR97, Including Novel Bacteriocins, as a Natural Alternative to Chemical Disinfectants

**DOI:** 10.3390/ph13100266

**Published:** 2020-09-23

**Authors:** Sam Woong Kim, Song I. Kang, Da Hye Shin, Se Yun Oh, Chae Won Lee, Yoonyong Yang, Youn Kyoung Son, Hee-Sun Yang, Byoung-Hee Lee, Hee-Jung An, In Sil Jeong, Woo Young Bang

**Affiliations:** 1 Gene Analysis Center, Gyeongnam National University of Science & Technology, Jinju 52725, Korea; swkim@gntech.ac.kr (S.W.K.); mole160104@naver.com (S.I.K.); nini1114@naver.com (D.H.S.); ks-sy0809@naver.com (S.Y.O.); 2 National Institute of Biological Resources (NIBR), Environmental Research Complex, Incheon 22689, Korea; chaewon326@korea.kr (C.W.L.); tazemenia@korea.kr (Y.Y.); sophy004@korea.kr (Y.K.S.); moeicy@korea.kr (H.-S.Y.); dpt510@korea.kr (B.-H.L.); 3 Department of Pathology, CHA Bundang Medical Center, CHA University, Seongnam 13496, Korea; hjahn@cha.ac.kr; 4 Center for Immune Cell Research, CHA Advanced Research Institute, Seongnam 13488, Korea

**Keywords:** AMP, antimicrobial activity, antiviral activity, bacteriocin, COVID-19, disinfectant, *Lactobacillus plantarum*, plantaricin

## Abstract

The recent pandemic of coronavirus disease 2019 (COVID-19) has increased demand for chemical disinfectants, which can be potentially hazardous to users. Here, we suggest that the cell-free supernatant from *Lactobacillus plantarum* NIBR97, including novel bacteriocins, has potential as a natural alternative to chemical disinfectants. It exhibits significant antibacterial activities against a broad range of pathogens, and was observed by scanning electron microscopy (SEM) to cause cellular lysis through pore formation in bacterial membranes, implying that its antibacterial activity may be mediated by peptides or proteins and supported by proteinase K treatment. It also showed significant antiviral activities against HIV-based lentivirus and influenza A/H3N2, causing lentiviral lysis through envelope collapse. Furthermore, whole-genome sequencing revealed that NIBR97 has diverse antimicrobial peptides, and among them are five novel bacteriocins, designated as plantaricin 1 to 5. Plantaricin 3 and 5 in particular showed both antibacterial and antiviral activities. SEM revealed that plantaricin 3 causes direct damage to both bacterial membranes and viral envelopes, while plantaricin 5 damaged only bacterial membranes, implying different antiviral mechanisms. Our data suggest that the cell-free supernatant from *L. plantarum* NIBR97, including novel bacteriocins, is potentially useful as a natural alternative to chemical disinfectants.

## 1. Introduction

Severe acute respiratory syndrome coronavirus 2 (SARS-CoV-2), responsible for the global pandemic of coronavirus disease 2019 (COVID-19), is the foremost concern among recent global health issues [1]. For prevention of this infection, disinfectants have been widely used—mainly because SARS-CoV-2, like other coronaviruses and enveloped viruses, is surrounded by a fragile outer lipid envelope, which makes it more susceptible to disinfectants than non-enveloped viruses such as rotavirus, norovirus, and poliovirus [2]. Accordingly, the pandemic of COVID-19 has led to a large surge in demand for disinfectants, especially chemical disinfectants such as alcohol- or chlorine-based formulas for the disinfection of hands or environmental surfaces [3,4,5]. Although chemical disinfectants are considered very effective, they could be hazardous to users if they are not properly handled; for example, alcohol-based disinfectants are flammable and can be harmful to humans if they enter the body [3]. For this reason, there is increasing interest in disinfectants based on natural products.

Lactic acid bacteria, traditionally used in fermented foods, have been considered as interesting resources to contribute to developing a safe alternative to biocides, which are potentially hazardous to humans, because they produce diverse antimicrobial substances and are seldom hazardous to humans [6,7]; most are approved by the U.S. Food and Drug Administration as GRAS (Generally Recognized as Safe). As typical antimicrobial substances, they secrete lactic acid with bacteriocins and antimicrobial peptides (AMPs), which are produced by most microbes [6,7]. In particular, bacteriocins, such as nisin, sakacin, plantaricin, and leucocin from lactic acid bacteria have been reported to have antibacterial activity against foodborne bacteria, such as *Escherichia coli*, *Salmonella enterica*, and *Listeria monocytogenes*, and thus many studies have highlighted their application as natural alternatives to artificial preservatives and antibiotics [6,8,9,10]. In addition, several bacteriocins have shown antiviral activities against pathogenic viruses such as poliovirus, herpes simplex virus, and influenza viruses [10,11,12]. Accordingly, the cell-free supernatant, including the bacteriocins and lactic acid, has potential as a natural alternative to chemical disinfectants, although there have been no attempts to apply it as a disinfectant as of yet. To the best of our knowledge, this report is the first that addresses these issues.

In this study, we first suggest that the cell-free supernatant from *Lactobacillus plantarum* NIBR97, a lactic acid bacterium isolated from kimchi, a Korean fermented food, could potentially be useful for disinfection against both pathogenic bacteria and viruses, mediated by bacteriocins as well as lactic acid. Through the genomic analysis of the NIBR97 strain, we discovered novel bacteriocins functioning as antibacterial and antiviral peptides. Our study will provide important information that will guide new strategies to replace chemical disinfectants with natural substances.

## 2. Results

### 2.1. Antibacterial Activity of Cell-Free Supernatant from L. plantarum NIBR97

**Figure 1:**
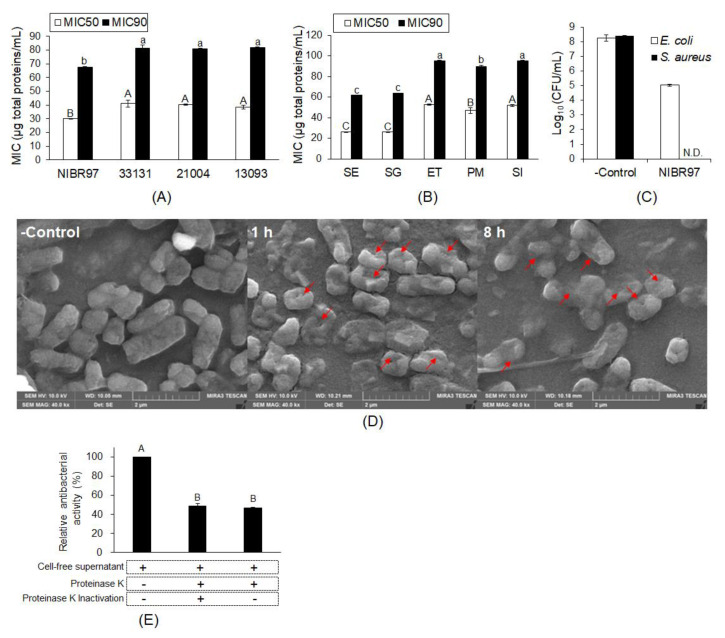
Antibacterial activity of cell-free supernatant from *Lactobacillus plantarum* NIBR97. Antibacterial activities of the cell-free supernatant from the *L. plantarum* strains NIBR97, KCTC33131, KCTC21004, and KCTC13093 were examined against *Salmonella* Enteritidis, whose MIC50s and MIC90s were determined (**A**). The MIC50s and MIC90s of the cell-free supernatant were determined against *Salmonella* Gallinarum (SG), *Edwardsiella tarda* (ET)*, Pasteurella multocida* (PM), and *Streptococcus iniae* (SI), as well as *S.* Enteritidis (SE) (**B**). For bactericidal activity, *Escherichia coli* and *Staphylococcus aureus* were treated with the cell-free supernatant (126.6 μg total proteins/mL) for 5 min, and then were counted to determine the titer (Log_10_ (colony-forming unit (CFU)/mL) and reduction rate (%) (**C**). For scanning electron microscopy, *S.* Enteritidis was treated without (control) or with the cell-free supernatant (70.8 μg total proteins/mL, MIC against *S.* Enteritidis) for 1 h and 8 h (**D**). The red arrows indicate the pores forming in the *Salmonella* membrane. (**E**) To investigate the effect of protease on the antibacterial activity of the cell-free supernatant, we added the proteinase K (100 µg/mL) to the cell-free supernatant at 70.8 μg total proteins/mL and the treated sample was used to examine its antibacterial activity against *S.* Enteritidis. In (**E**), the plus (+) mark indicates the treatment of cell-free supernatant or proteinase K, whereas the minus (−) mark does no treatment, and the proteinase K was inactivated at 80 °C for 10 min (+) or not (−). The different letters (A, B, C, a, b and c) in the graphs ((**A**), (**B**), (**C**) and (**E**)) represent significant differences (*p* < 0.05) and in (**A**) and (**B**), the capital (A, B and C) and small letters (a, b c) indicate the significant differences in MIC50 and MIC90 data, respectively.

*Lactobacillus plantarum* NIBR97 were screened from kimchi as a strain with superior antibacterial activity, and its cell-free supernatant was further used for the examination of antibacterial activity, as shown in Figure 1. The minimum inhibitory concentrations (MIC50 and MIC90) were determined as 30.04 and 67.43 μg total proteins/mL against *Salmonella enterica* Serovar Enteritidis (*S*. Enteritidis), respectively, which indicates significantly higher antibacterial activity than the three *Lactobacillus plantarum* strains, KCTC33131, KCTC21004, and KCTC13093, with higher MIC50 and MIC90 values than NIBR97 (Figure 1A and Figure S1A). The cell-free supernatant also showed MIC50s and MIC90s against *Salmonella* Gallinarum, *Edwardsiella tarda*, *Pasteurella multocida*, and *Streptococcus iniae* (Figure 1B and Figure S1B), implying antibacterial activity against broad pathogenic bacteria. In addition, when *Escherichia coli* and *Staphylococcus aureus* were treated with the cell-free supernatant for 5 min, they showed a reduction of at least 99.9% (≥3 log_10_) of the total count in the original inoculum (Figure 1C), indicating bactericidal activity and potential as a disinfectant.

In order to prove the antibacterial activity against pathogenic bacteria with the cell-free supernatant from *L. plantarum* NIBR97, we observed the *S*. Enteritidis treated with the cell-free supernatant using scanning electron microscopy (SEM). As shown in Figure 1D, the SEM images revealed that the cell-free supernatant effectively caused the Salmonella death via pore formation by cellular penetrating peptides, as is the case for typical AMPs [13]. Furthermore, when the cell-free supernatant was treated with proteinase K, its antibacterial activity against *S.* Enteritidis decreased by about 50% compared with the control without the proteinase K treatment (Figure 1E). Therefore, we suggest that proteins or peptides play major roles for the antibacterial activities of cell-free supernatant from *L. plantarum* NIBR97.

### 2.2. Antiviral Activity of Cell-Free Supernatant from L. plantarum NIBR97

**Figure 2:**
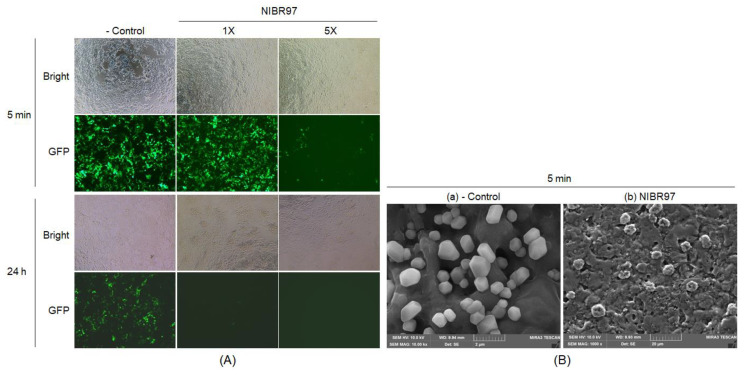
Antiviral activity of cell-free supernatant from *L. plantarum* NIBR97. (**A**) For fluorescence microscopy, we treated GFP-labeled lentiviruses with the cell-free supernatant for 5 min and 24 h, and then were infected in HEK-293T human host cells. The 1× and 5× correspond to the concentrations treated to the lentiviruses, 79.15 and 395.75 μg total proteins/mL, respectively. The bright-field images (Bright) indicate the HEK-293T cells, and the green signals in the fluorescent images (GFP) represent the GFP-labeled lentiviruses. (**B**) For scanning electron microscopy, the GFP-labeled lentiviruses were treated without (**a**) or with the cell-free supernatant (395.75 μg total proteins/mL) (**b**) for 5 min.

To assess its antiviral activity, the cell-free supernatant from *L. plantarum* NIBR97 was exposed to green fluorescent protein (GFP)-labeled lentiviruses, based on human immunodeficiency virus (HIV), which causes acquired immunodeficiency syndrome (AIDS), for 5 min and 24 h, as shown in Figure 2. When the GFP-labeled lentiviruses, treated with the cell-free supernatant, infected the HEK-293T cells (human host cells), they were observed by fluorescence microscopy to decrease dose- and time-dependently within the host cells (Figure 2A, the GFP images) without any cytotoxic effect on the human host cells (Figure 2A, the Bright images). SEM also confirmed its antiviral activity by showing that the cell-free supernatant effectively causes lentiviral lysis through the collapse of envelopes after 5 min (Figure 2B). In addition, when the human influenza A virus subtype H3N2 (A/H3N2) was treated with the cell-free supernatant, it showed a reduction of at least 99.5% of the total count of its original inoculums, which increased until 99.999% with treatment time (Table 1). These results indicate that the cell-free supernatant from *L. plantarum* NIBR97 has superior antiviral activity, as well as potential as a disinfectant.

### 2.3. Discovery of Novel Bacteriocins by the Genomic Analysis of L. plantarum NIBR97

**Figure 3:**
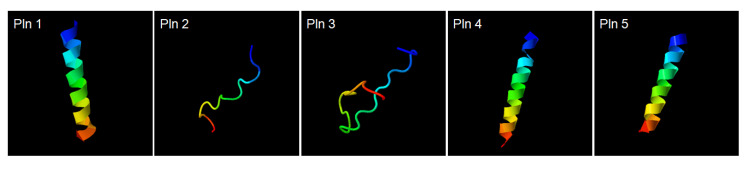
Structural models of plantaricins. Pln 1, 2, 3, 4, and 5 comprise the amino acid sequences VLGSLIGSVGIGVLSSLAARYK, IYPEKQPEEPVRR, KKSRRCQVYNNGMPTGMYTSC, PIVREPFKAMAVGIILAVMSGLLVT, and KAKKRFLRNRLSQQARKARTK, respectively. Pln 1, 4, and 5 form helix structures, and Pln 2 and 3 form random coil structures. The structures of Pln 1, 2, 3, 4, and 5 were predicted by the automated I-TASSER server (https://zhanglab.ccmb.med.umich.edu/I-TASSER/).

Analysis of the whole-genome sequence for the *L. plantarum* NIBR97 was carried out by the PacBio RS II (Pacific Biosciences, Menlo Park, CA, USA) sequencing platform to identify the AMPs from the NIBR97. The NIBR97 genome identified from de novo assembly was composed of a single circular bacterial chromosome and four plasmids, containing 2927 predicted open reading frames (ORFs), 68 tRNAs, and 16 rRNAs (Table 2 and Figure S2). Among the ORFs, 10 were identified to encode homologous proteins with known AMPs via an NCBI (National Center for Biotechnology Information) homology BLAST (Basic Local Alignment Search Tool) (Table S1). Furthermore, their expression in *L. plantarum* NIBR97 was confirmed by the transcriptomic data (Table S2). In detail, the five ORFs—orf02155, orf02163, orf02164, orf02421, and orf00645—were found to have 100% identities with plantaricin N, F, and E, as well as bacteriophage holing and lysozyme, known previously as AMPs from the *Lactobacillus* genus (Table S1). Five ORFs—orf00467, orf01336, orf01363, orf01599, and orf01790—which were previously uncharacterized until now, were discovered in this study to consist of amino acid sequences with high positives (>60%) with AMPs undiscovered in *L. plantarum* strains: grammistin Pp3, indolicidin, bactofencin A, hymenochirin-5B, and latarcin-2a, (Table S1). Thus, we herein designated the AMPs as novel bacteriocins called plantaricin (Pln) 1, 2, 3, 4, and 5 (Table S1). Interestingly, their structural models revealed that the three plantaricins—Pln 1, 4 and 5—form helix structures, and the two plantaricins—Pln 2 and 3—form random coil structures (Figure 3), similar to typical AMPs [14,15], implying that they may have antibacterial activities.

### 2.4. Antibacterial and Antiviral Activities of Plantaricins from L. plantarum NIBR97

**Figure 4:**
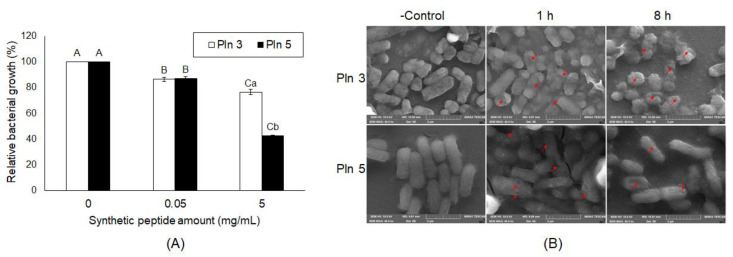
Antibacterial activity of plantaricin 3 and 5 against *S*. Enteritidis. Pln 3 and 5 were synthesized according to the amino acid sequences in Figure 3, and further examined for their antibacterial activity against *S*. Enteritidis (**A**). The *y*-axis and different letters (A, B, C, a and b) in the graphs represent the relative bacterial growth (%) and significant differences (*p* < 0.05), respectively. In (**A**), the capital (A, B and C) and small letters (a and b) indicate the significant differences between different concentrations (0-5 mg/mL) and the ones between Pln 3 and 5, respectively. (**B**) For scanning electron microscopy, *S.* Enteritidis was treated without or with synthetic Pln 3 or 5 (5 mg/mL) for 1 h and 8 h. The red arrows indicate the pores forming in the *Salmonella* membrane.

To confirm whether the five Plns function as AMPs, we assessed their synthetic peptides for antibacterial activity against *Salmonella* Typhimurium (Figure S3). Among them, Pln 5 exhibited the highest antimicrobial activity, showing the lowest MIC50 compared with others, whereas Pln 4 showed the lowest antimicrobial activity (Figure S3). In addition, the Pln 3 and 5 were identified to inhibit the growth of *Salmonella* Enteritidis (Figure 4A) and were observed by SEM to effectively cause cellular lysis by damaging the membrane of *S*. Enteritidis via pore formation (Figure 4B), as did the cell-free supernatant from *L. plantarum* NIBR97 (Figure 1D).

**Figure 5:**
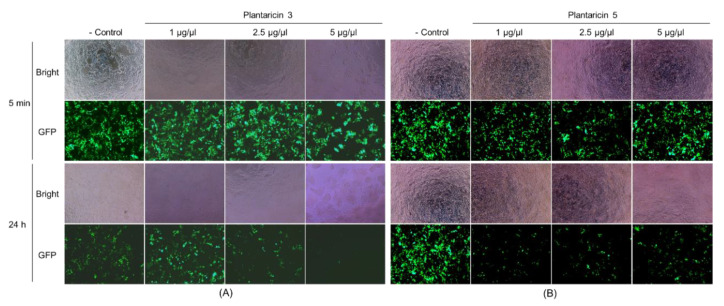
Fluorescence micrographs of HEK-293T cells infected with GFP-labeled lentiviruses treated with synthetic Pln 3 and 5. The lentiviruses were treated without (control) or with the synthetic peptides (1 to 5 μg/μL) Pln 3 (**A**) and 5 (**B**) for 5 min or 24 h, and then the HEK-293T human host cells were infected. The bright-field images (Bright) indicate the HEK-293T cells, and the green signals in the fluorescent images (GFP) represent the GFP-labeled lentiviruses.

**Figure 6:**
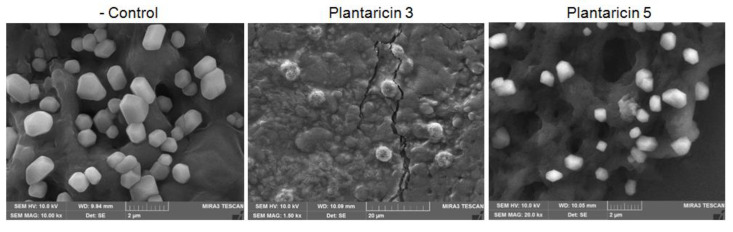
Scanning electron micrographs of the GFP-labeled lentiviruses treated with synthetic Pln 3 and 5. The lentiviruses were treated without or with the synthetic peptides at 5 μg/μL for 24 h.

The synthetic Pln 3 and 5 were further examined for antiviral activity against GFP-labeled lentiviruses. The synthetic peptides exhibited a cytotoxicity on the human host cells when the lentiviruses were treated with 5 μg/μL of synthetic peptides, but not with ≈2.5 μg/μL of synthetic peptides (Figure 5, the Bright images). The fluorescence microscopy revealed that the lentiviruses decreased considerably within the host cells when they were treated with the Pln 3 or 5 for 24 h, but not for 5 min (Figure 5, the GFP images). This suggests that Pln 3 and 5 can considerably suppress viral infection in host cells. Interestingly, SEM revealed that Pln 3 effectively caused lentiviral lysis through the collapse of the envelopes (Figure 6), as the cell-free supernatant did (Figure 2B), whereas Pln 5 did not (Figure 6). This implies that Pln 3 and 5 may exert their antiviral role through different mechanisms. 

## 3. Discussion

*Lactobacillus plantarum* is one of the most widespread lactic acid bacteria species and is largely used for the production of fermented products of animal and plant origin [16]. Moreover, some strains are known to produce several natural antibacterial substances, such as bacteriocins, organic acids (mainly lactic and acetic acid), and hydrogen peroxide [17,18], and thus many studies have highlighted their application as preservatives and antibiotics [6,8,9,10]. Here, we investigated their potential as a natural alternative to chemical disinfectants.

In this study, the NIBR97 strain was screened from kimchi, a Korean fermented food, and its cell-free supernatant was identified to have higher antibacterial activity against *Salmonella* bacteria than other *L. plantarum* strains (Figure 1A), as well as possessing antibacterial activities against a broad range of pathogenic bacteria (Figure 1B). It exhibited significant disinfection activities against the human pathogens influenza A virus H3N2, *Escherichia coli*, and *Staphylococcus aureus*, reducing them by at least 99.9% of the total count of their original inoculums within 30 min (Figure 1C and Table 1). These results indicate that the cell-free supernatant from *L. plantarum* NIBR97 has potential as a natural disinfectant, and thus further investigations were performed to identify the antimicrobial substances, such as AMPs, in the NIBR97 strain.

AMPs are small peptides composed of 10 to 40 amino acids, which cause microbial membrane modification via either pore formation by cell-penetrating property through a barrel stave or a toroidal pore mechanism, or through a non-pore carpet-like mechanism [13,19]. Our scanning electron micrographs of *S*. Enteritidis showed clearly that the cell-free supernatant from the NIBR97 formed a pore on the *Salmonella* surface (Figure 1D), as do typical AMPs [13]. Proteinase K treatment of the cell-free supernatant led to a considerable decrease in its antibacterial activity against both *S*. Enteritidis and *S*. Gallinarum (Figure 1E). Thus, these results confirm that the antibacterial activities of the cell-free supernatant from the NIBR97 are mediated mainly by its proteins or peptides, functioning as AMPs. The scanning electron micrographs of GFP-labeled lentivirus showed that the cell-free supernatant causes lentiviral lysis through envelope collapse (Figure 2A), but it was unclear whether the AMPs were involved in the envelope collapse of the virus.

Finally, to identify AMPs from the NIBR97 strains, we performed whole-genome sequencing, which revealed that the 10 ORFs encoded AMPs, including known forms (plantaricin E, F, N; bacteriophage holin; lysozyme) and novel forms (Pln 1 to 5 (Table S1)). In the case of the known AMPs, plantaricin E, F, and N are bacteriocins produced in *Lactobacillus plantarum* C11 [20]; holin, produced by bacteriophages, triggers and controls the degradation of the cell wall of the host bacteria [21]; and lysozyme functions as 1,4-beta-N-acetylmuramidase, an antimicrobial enzyme, and has been found mainly in *Lactobacillus rhamnosus* strains [22]. Interestingly, five ORFs were discovered as novel bacteriocins in this study (Figure 3) and were designated as Pln 1, 2, 3, 4, and 5. They were further confirmed as being expressed in the NIBR97 strain through transcriptomic sequencing (Table S2), and even their synthetic peptides exhibited antibacterial activity against *Salmonella* Typhimurium (Figure S3). The synthetic Pln 3 and 5 also inhibited the growth of *S.* Enteritidis and effectively caused cellular lysis through damage to the *Salmonella* membrane via pore formation (Figure 4), suggesting that they function as AMPs. However, the synthetic Plns showed overall lower antibacterial activities than antibiotics such as octenidine when their MICs were compared with each other (Figure S3) [23]. This is presumably because the Plns were not synthesized on the basis of complete amino acid sequences for the optimal antibacterial activity but were done on the basis of minimal sequences for the activity; thus, it is further necessary to identify the mature peptide sequence responsible for the optimal antibacterial activity, following the signal peptide cleavage. Moreover, Pln 3 and 5 were identified to suppress lentiviral infection in human host cells (Figure 5). Collectively, these results suggest that the cell-free supernatant from *L. plantarum* NIBR97 may include AMPs, such as Pln 3 and 5, exhibiting antibacterial and antiviral activities. However, Pln 3 and 5 were observed by SEM to act differentially in the suppression of viral infection; Pln 3 had a significant effect on the viral shape through the collapse of the viral envelope, which suggests that it may cause direct damage to the envelope. In contrast, Pln 5 had little effect on it (Figure 6), which implies that it may interfere with the interaction between viruses and host cells [24,25].

Noticeably, Pln 3 and 5 suppressed viral infection when used against lentivirus for 24 h, but not for 5 min (Figure 5), which indicates that long exposure is required for their antiviral role. Although the Plns exhibited low antibacterial activities as mentioned above, during long expose (i.e., 24 h), they may also contribute significantly to the antibacterial activities of cell-free supernatant, together with other AMPs discovered by genomic analysis of NIBR97, which is strongly supported by the proteinase K treatment leading to a considerable decrease (>50%) in antibacterial activity of the cell-free supernatant (Figure 1E). Furthermore, this is confirmed by Figure S4—the cell-free supernatant from the *E. coli* Top10 strain (Invitrogen, Carlsbad, CA, USA), harboring each *Pln* gene cloned, showed significant antibacterial activities against both Gram-negative and Gram-positive bacteria, whereas very little antibacterial activity was detected in the negative control, that is, treatment with the cell-free supernatant from the strain without the *Pln* genes (Figure S4). Meanwhile, the disinfection activity of the cell-free supernatant during short exposures (i.e., within 30 min), as shown in Figure 1C and Table 1, was presumably because the lactic acid may have functioned as a disinfectant during the short exposure. This is supported by the data, showing that the cell-free supernatant contained considerable lactic acids (≈2%) when the NIBR97 strain was cultured in the de Man, Rogosa and Sharpe (MRS) medium, consisting of 5% solutes and 95% water, for 24 h (Figure S5), and by a previous report stating that they induce sudden severe acid stress, leading to a shock of oxidative stress and resulting in the destabilization of the bacterial membrane [26]. Therefore, the cell-free supernatant may exert its role as a disinfectant, mainly through lactic acid during short exposure (i.e., within 30 min), while it does so through an integrated effect between the lactic acid and the various AMPs during long exposure (i.e., 24 h).

## 4. Materials and Methods

### 4.1. Materials

As susceptible bacteria to AMPs, *S.* Enteritidis, *S*. Gallinarum, *Edwardsiella tarda, Pasteurella multocida,* and *Streptococcus iniae* were obtained from Dr. Jin Hur (Chonbuk National University, Iksan, Korea) and Dr. Tae Sung Jung (Gyeongsang National University, Jinju, Korea). The *Lactobacillus plantarum* strains KCTC33131, KCTC21004, and KCTC13093, as well as the susceptible bacteria *Escherichia coli* ATCC 10536 and *Staphylococcus aureus* ATCC 6538, were purchased from KCTC (Korean Collection for Type of Cultures, Daejeon, Korea). The human influenza A/H3N2 was provided by the Korea Centers for Disease Control and Prevention (KCDC, Chungcheongbuk-do, Korea). The plasmids for lentiviral packaging (two packaging vectors, pRSV-Rev and pCgpV, and an envelope vector, pCMV-VSV-G) and for a positive control of transduction (pSIH1-H1-siLUC-copGFP) were purchased from Cellbiolab (San Diego, CA, USA) and System Biosciences (Palo Alto, CA, USA), respectively. The five synthetic peptides—plantaricin 1 to 5—were purchased from Cosmogenetech Inc. (Seoul, Korea).

### 4.2. Analysis of the Minimal Inhibitory Concentration (MIC50 and MIC90)

*L. plantarum* NIBR97 was incubated at 37 °C for 24 h in an MRS liquid medium (10 g/L peptone, 8 g/L meat extract, 4 g/L yeast extract, 20 g/L d(+)-glucose, 2 g/L dipotassium hydrogen phosphate, 5 g/L sodium acetate trihydrate, 2 g/L triammonium citrate, 0.2 g/L magnesium sulfate heptahydrate, and 0.05 g/L manganous sulfate tetrahydrate). The cultural broth was centrifuged for 20 min at 2000× *g*, and the centrifugal supernatant was collected and then sterilized by a 0.22 μm filtration. The sterilized fluid was either applied directly for the examination of antimicrobial activity or fractionated and stored at −80 °C until use. The assessment of antimicrobial activity on a microtiter plate was performed by some modification of the dilution assay of Wiegand et al. [27]. The MIC50 and MIC90 were expressed as total proteins equivalent (μg) per volume (mL) of the sample, and the effect of proteinase K treatment was examined by a previously described procedure [28].

### 4.3. Measurement of Bactericidal Activity

The susceptible bacterial strains *Escherichia coli* ATCC 10536 and *Staphylococcus aureus* ATCC 6538 were adjusted into 1.5 to 5.0 × 10^8^ CFU/mL after pre-culture, and 10% sucrose was used as an interfering agent, 0.25 M KH_2_PO_4_ (pH 7.2) was used as a neutralizing agent, and 20 mg/mL proteinase K was used to degrade the AMPs. For the bactericidal activity assay, we mixed 100 μL of prepared susceptible bacterial solution, 100 μL 10% sucrose, and 800 μL cell-free supernatant (126.6 μg total proteins/mL) from *L. plantarum* NIBR97 and reacted the mixture at 20 °C for 5 min. An aliquot (100 μL) of the reaction solution was mixed with 800 μL 0.25 M KH_2_PO_4_ (pH 7.2), 5 μL proteinase K, and 100 μL distilled water, and then reacted at 20 °C for 5 min. The surviving cells were counted by serial dilution of the treated solution and incubation on an Luria-Bertani (LB) plate.

### 4.4. Scanning Electron Microscopy (SEM)

The *S.* Enteritidis was treated with the cell-free supernatant (70.8 μg total proteins/mL, MIC against *S.* Enteritidis) from *L. plantarum* culture or the synthetic peptides, Pln 3 (1 μg/μL) or Pln 5 (1 μg/μL), for 0, 1, and 8 h, and the lentivirus was assessed with the cell-free supernatant (15.8 μg total proteins/mL) for 5 min and with the synthetic peptides Pln 3 (5 μg/μL) or Pln 5 (5 μg/μL) for 24 h. The treated bacteria and viruses were observed by a scanning electron microscope according to previously described procedures [28].

### 4.5. Antiviral Analysis Against Influenza A/H3N2

For the antiviral test, we co-incubated 0.1 mL of the A/H3N2 soup (2–4 × 10^5^ viruses/μL) with 0.9 mL of the cell-free supernatant (142.5 μg total proteins/mL) for 10 min, 30 min, and 18 h at 25 °C. After the co-incubation, the cell-free supernatant-A/H3N2 mixture was 10-fold serially diluted to infect Madin–Darby canine kidney (MDCK) cells (3 × 10^4^ cells per well) and, thereafter, the cell culture infectious dose (CCID50) and the viral reduction were determined by cytopathic effect (CPE) and plaque assays, as previously described [29]. 

### 4.6. Antiviral Analysis Against GFP-Labeled Lentivirus

For the production of GFP-labeled lentivirus, we transfected 5 μg of pRSV-Rev, 5 μg of pCMV-VSV-G, 5 μg of pCgpV, and 15 μg of pSIH1-H1-siLUC-copGFP plasmids into HEK-293T cells (6 × 10^6^ cells per well) using lipofectamine 2000 (Invitrogen, Carlsbad, CA, USA). The lentiviral supernatants were harvested 72 h after transfection, filtered through Millex-GP 0.45 µm filters (Millipore, Schwalbach, Germany), and concentrated using Retro-Concentin Retroviral Concentration Reagent (System Biosciences, Palo Alto, CA, USA). The titer of lentiviruses was measured with a QuickTiter Lentivirus Titer Kit (Cellbiolabs, San Diego, CA, USA) and stored at −80 °C.

For the anti-viral test, we co-incubated 2 μL of lentivirus soup (2.8 × 10^6^ lentiviruses/μL) with 2 μL of test sample for 5 min and 24 h at 25 °C. After the co-incubation, 2 μL from the total 4 μL of the test sample–lentivirus mixture was infected in HEK-293T cells (1 × 10^4^ cells per well). Expression of the copGFP protein was observed at day 3 after infection with a Zeiss 510 fluorescence microscope (Carl Zeiss Co., Oberkochen, Germany).

### 4.7. Analysis of the Genome

Genomic analysis of *L. plantarum* NIBR97 was performed by previously described procedures. In detail, genomic DNA from the NIBR97 was extracted and sequenced by previously described procedures [28]. De novo assembly and putative gene coding sequences (CDSs) from the assembled contigs was performed by the hierarchical genome assembly process (HGAP, Version 3) workflow [30] and the bacterial genome was checked by MUMmer 3.5 [31], identifying that the genome comprises a single circular DNA chromosome of 3,022,780 bp with four plasmids by trimming one of the self-similar ends for manual genome closure (Table 2). Putative gene coding sequences (CDSs) from the assembled contigs were identified by Glimmer v3.02 [32], and the obtained ORFs were examined by Blastall alignment (http://www.ncbi.nlm.nih.gov/books/NBK1762). Gene ontology annotations of the ORFs were assigned by Blast2GO software [33]. In addition, ribosomal RNAs and transfer RNAs were separated by RNAmmer 1.2 and tRNAscan-SE 1.4 [34,35]. Finally, the whole-genome sequence data were deposited as Sequence Read Archive (SRA) data in GenBank (SRA no., SRR12344691; BioProject no., PRJNA647132).

### 4.8. Statistical Analysis

The mean values were separated by the probability difference option according to significant differences. The results are exhibited as least square means with standard deviations. Duncan’s multiple range tests (MRT) were applied for verification of significant differences (*p* < 0.05) between sample types. All the analyses were performed by the SAS statistical software package (version 9.1, SAS Inst., Inc., Cary, NC, USA), for which differences were considered significant at *p* < 0.05.

## 5. Conclusions

Together, our data showed that the cell-free supernatant from *L. plantarum* NIBR97, producing novel bacteriocins, has superior antibacterial and antiviral activities during both short and long exposures, which suggests that it is potentially useful as a natural material to completely or partially replace chemical disinfectants.

## Figures and Tables

**Table 1:** Disinfection activity of the cell-free supernatant from *L. plantarum* NIBR97 against A/H3N2.

Treatments ^1^	10 min ^1^		30 min ^1^		18 h ^1^	
	Titer ^2^	Reduction ^3^	Titer ^2^	Reduction ^3^	Titer ^2^	Reduction ^3^
Water	5.66	0	5.45	0	5.34	0.21
NIBR97	3.27	99.594	<0.51	>99.999	<0.51	>99.999

^1^ The A/H3N2 viruses were treated with water, a negative control, or the cell-free supernatant (NIBR97) for 10 min, 30 min, and 18 h; ^2^ and ^3^ indicate the viral titer (log_10_CCID50) and reduction (%), respectively.

**Table 2:** Summary of the de novo genome assembly of *L. plantarum* NIBR97.

Items	Contig 1	Contig 2	Contig 3	Contig 4	Contig 5
Form	A circular chromosome	A circular plasmid	A circular plasmid	A linear plasmid	A linear plasmid
Length ^1^	3,022,780	61,378	32,520	7394	6876
GC ^2^	44.74	39.22	39.59	34.33	35.67
ORF ^3^	2816	60	32	10	9
rRNA ^4^	16	0	0	0	0
tRNA ^5^	68	0	0	0	0

^1^ and ^2^ indicate the length (bp, base pair) and GC (guanine-cytosine) contents (%) of contig in the form, respectively; ^3^, ^4^, and ^5^ represent the number of predicted open reading frames (ORFs), rRNA, and tRNA, respectively.

## Supplementary Materials

The following are available online at https://www.mdpi.com/1424-8247/13/10/266/s1, Figure S1. Antibacterial activity of cell-free supernatant from *L. plantarum* NIBR97. Figure S2. Overall features of the *L. plantarum* NIBR97 genome (contig 1) and plasmids (contig 2 to 5). Figure S3. Antibacterial activity of synthetic plantaricins identified from the *L. plantarum* NIBR97 genome. Figure S4 Antibacterial activity of the cell-free supernatant from *E. coli.* Top10 strain, harboring each *Pln* gene. Figure S5. The content of lactic acid in the cell-free supernatant from *L. plantarum* NIBR97. Table S1. Identification of ORFs predicted as antimicrobial peptides (AMPs) from the genome assembly data of *L. plantarum* NIBR97. Table S2. Transcriptomic analysis results of AMPs from *L. plantarum* NIBR97.
