# Supplementary material for: Potential of Cell-Free Supernatant from Lactobacillus plantarum NIBR97, Including Novel Bacteriocins, as a Natural Alternative to Chemical Disinfectants
Source: Pharmaceuticals (Basel). 2020 Sep 23;13(10):266. doi: 10.3390/ph13100266 (PMC7650660; doi:10.3390/ph13100266)
Supplement: Supplementary file 1 [file pharmaceuticals-13-00266-s001.pdf]

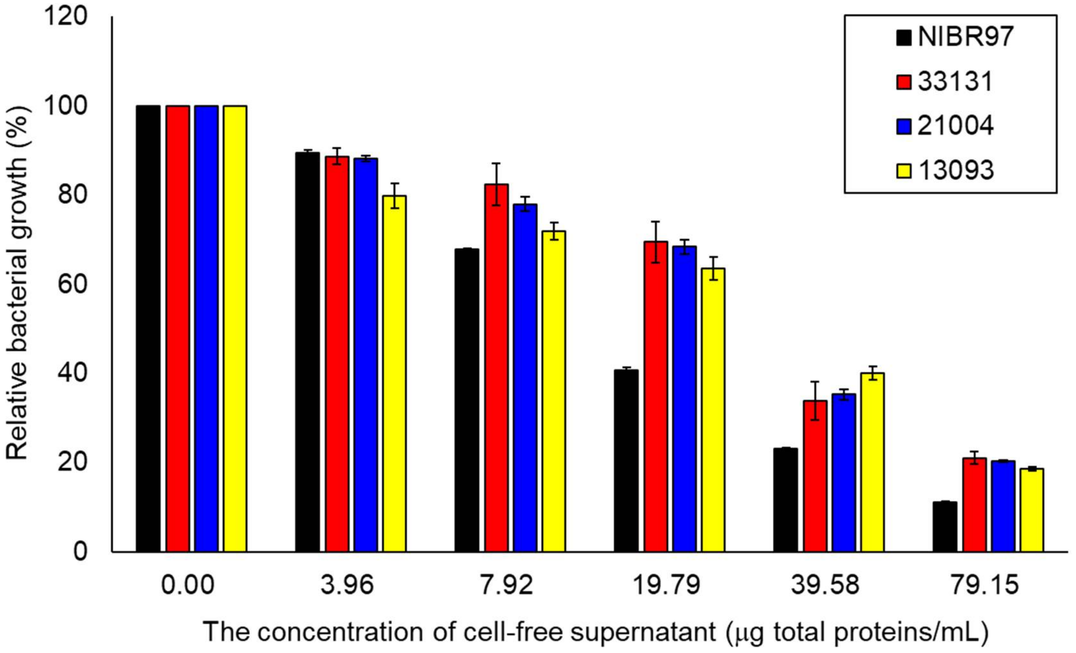

(A)

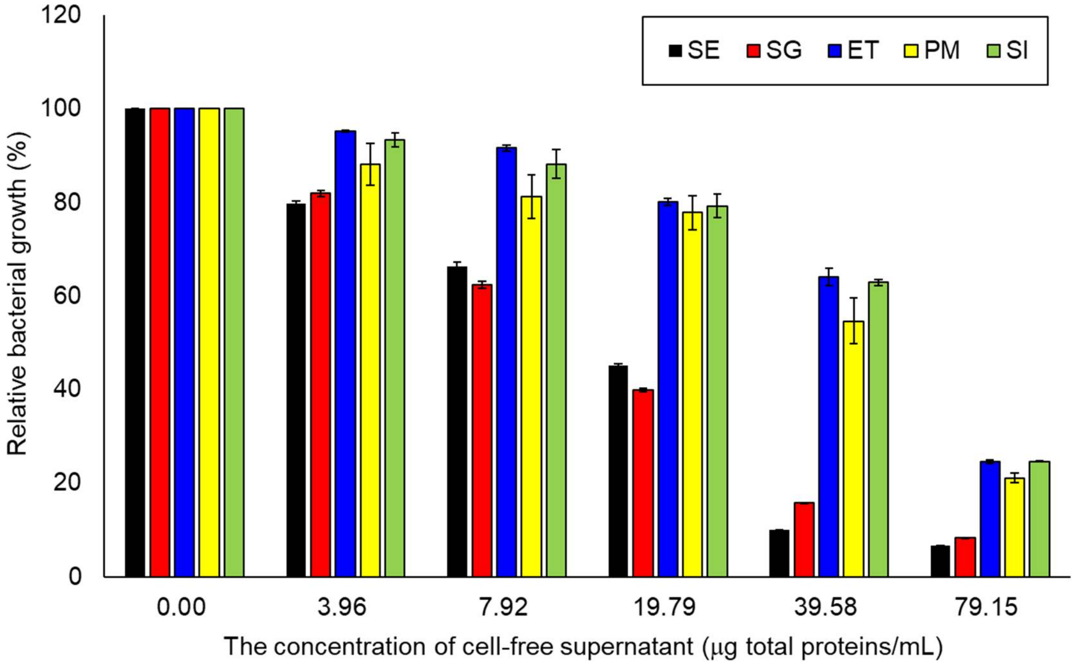

(B)

Figure 1. Antibacterial activity of cell-free supernatant from *L. plantarum* NIBR97. Antibacterial activities of the cell-free supernatant from the *L. plantarum* strains NIBR97, KCTC33131, KCTC21004 and KCTC13093 were examined against *S.* Enteritidis (A). The antimicrobial activity of cell-free supernatant from the NIBR97 were further examined against various susceptible bacteria, *Salmonella* Gallinarum (SG), *Edwardsiella tarda* (ET), *Pasteurella multocida* (PM) and *Streptococcus iniae* (SI), as well as *S. Enteritidis* (SE) (B). The Y axis in the graphs represents the relative growth of pathogenic bacteria (SE, SG, ET, PM or SI).

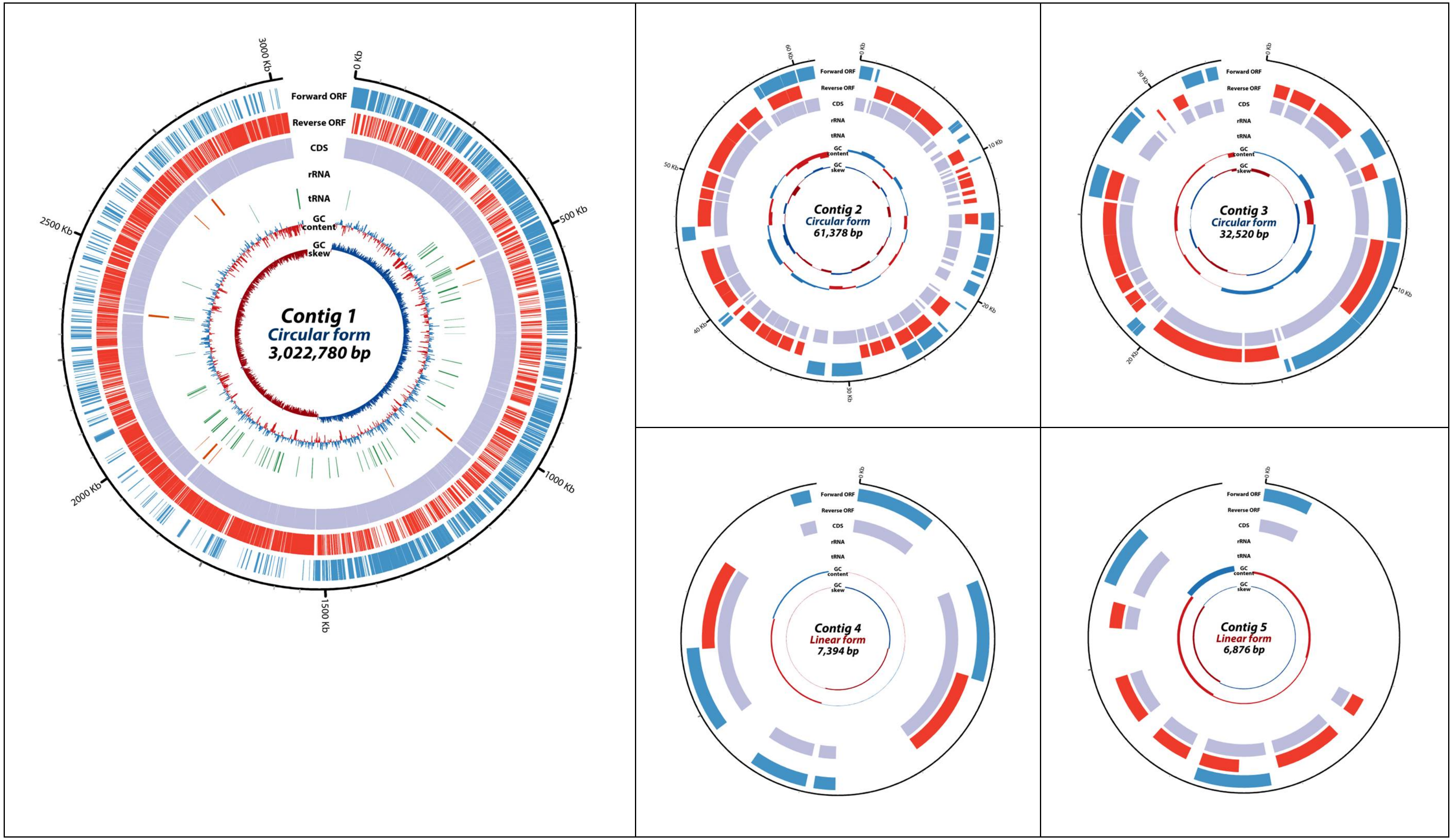

Figure 2. Overall features of the *L. plantarum* NIBR97 genome (Contig 1) and plasmids (Contig 2 to 5). The outer scale indicates the coordinates in base pairs. The open reading frames (ORF) is shown on the first two rings; first ring (blue) is the forward ORF and second ring (red) is the reverse ORF. The third and fourth circle shows the ORF which colored by gene annotation; third ring is forward ORF and fourth ring is reverse ORF. The fifth and sixth circle shows rRNA(green) and tRNA genes (orange). The next circle shows the GC content values. Purple and deep yellow colors indicate positive and negative sign, respectively. The inner-most circle shows GC skew, light green indicating negative values whereas deep orange for positive values.

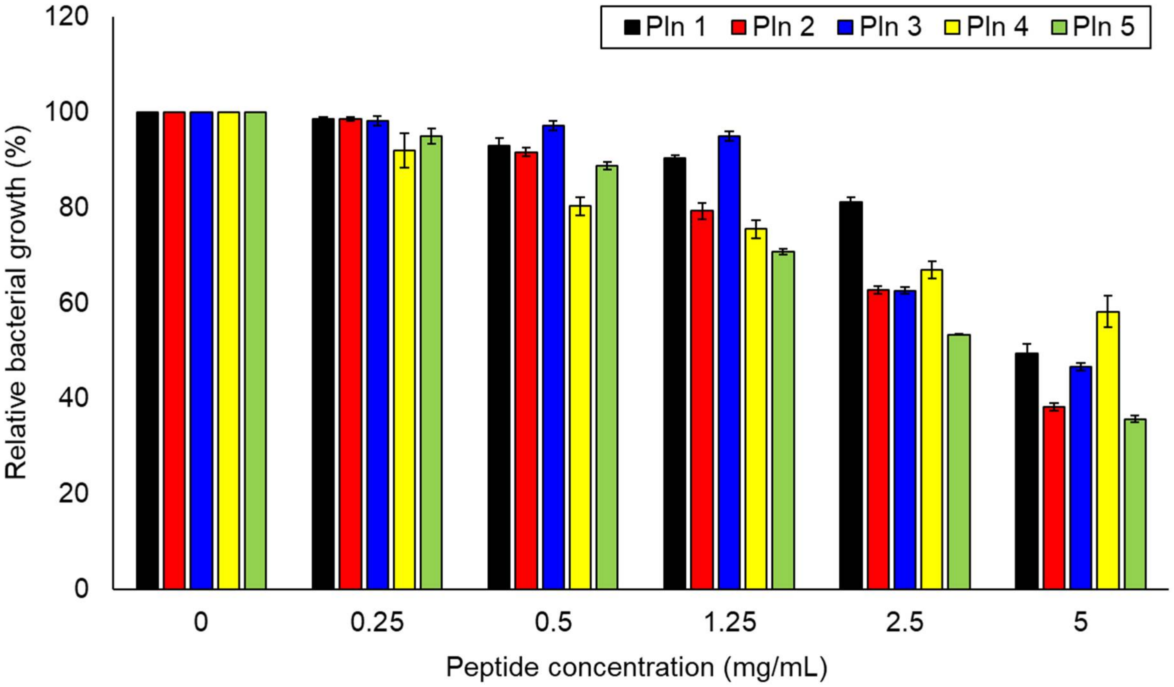

Figure 3. Antibacterial activity of synthetic Plantaricins identified from the *L. plantarum* NIBR97 genome. Pln 1, 2, 3, 4 and 5 were synthesized according to the amino acid sequences in Figure 3, and further examined for their antibacterial activity against a Salmonella enterica serotype, *S. Typhimurium*, whose MIC50s were determined as 5.48, 4.84, 5.17, 5.65 and 4.45 mg/mL, respectively. The Y axis in the graphs represents the relative growth of *S. Typhimurium.*

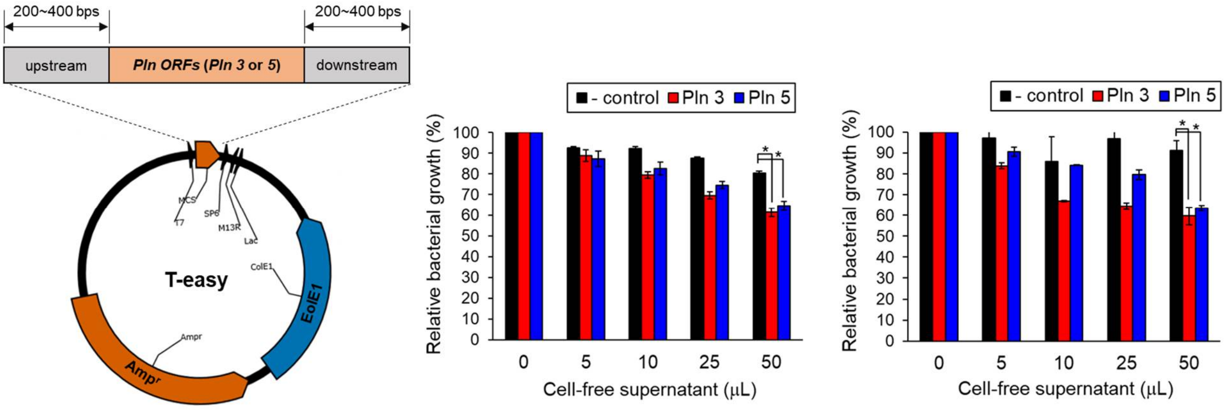

Figure 4. Antibacterial activity of the cell-free supernatant from *E. coli.* Top10 strain, harboring each *Pln* gene. The *E. coli.* Top10 strain (Invitrogen, Carlsbad, CA, USA), harboring each *Pln* gene, including its own intact promoter (upstream) and transcriptional terminator (downstream), was cloned into the pGEM®-T Easy Vector (Promega, Madison, WI, USA) (A) and its cell-free supernatant was used to examine antibacterial activities against a Gram-negative bacterium (pathogenic *E. coli*) (B) and a Gram-positive bacterium (*B. cereus*) (C). The Y axis and the * in the graphs represent the relative growth (%) of pathogenic bacteria and significant differences ($p < 0.05$) between - control and *Plns*, respectively.

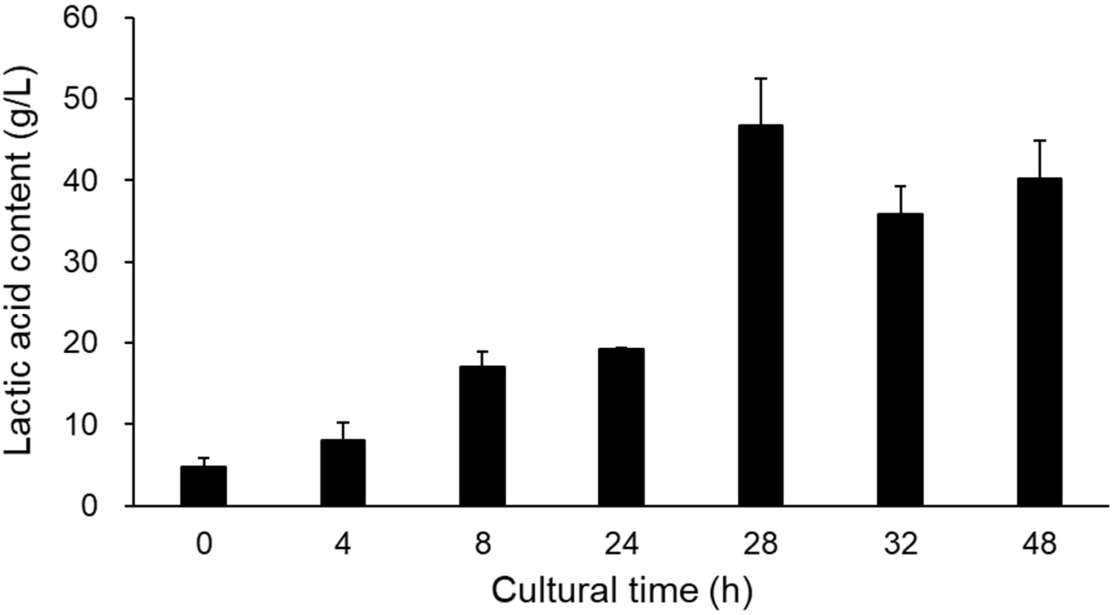

Figure 5. The content of lactic acid in the cell-free supernatant from *L. plantarum* NIBR97. *L. plantarum* NIBR97 was inoculated in MRS medium and cultured at 37 °C for the indicated time (X axis) in the graph to obtain the cell-free supernatant. Finally, the content of lactic acid in the cell-free supernatant was determined by thin layer chromatography (TLC) using the TLC silica gel 60 F254 Merck, Darmstadt, Germany) with nitromethane: 1-propanol: water (2:5:1.5, v/v/v) as a developing solvent and lactic acid (Sigma-Aldrich, St Louis, MO, USA) as a standard chemical. The Y axis in the graph represents the content of lactic acid (g/L) in the cell-free supernatant.

Table 1. Identification of ORFs predicted as AMPs from the genome assembly data of *L. plantarum* NIBR97. The AMPs were identified by NCBI blast (https://blast.ncbi.nim.nih.gov/).

No	ORF name	Peptide prediction	A.A. No	Identity
1	ORF00467	MGTIGQLVLAYIAVLGFGLIINIPHQAL<br>NVAGWIGTLTWGGYLIVQAFDGGVV<br>LGSLIGSVGIGVLSSLAARYKKMPAIIF<br>NIPSLVSFVPGSQAYQMVRNFALGNY<br>REAVSFTLQVIMITGAIALGFLLAELL<br>NRLIAFCIRQWRLHRLES	152	> CAMPSQ1094 Grammistin Pp3<br>Length=25<br>Score = 22.3 bits (46), Expect = 5.8<br>Identities = 12/22 (55%), Positives = 15/22 (68%), Gaps = 1/22 (5%)<br>> Renamded as plantaricin 1
2	ORF01336	MISLLEKHPNKITPHIPIEKPKKQLPQR<br>FPVKTPPENPQINPEKIYPEKQPEEPVR<br>RD	58	> DBAASP_4483|4833|Indolicidin<br>Length=13<br>Score = 25.4 bits (54), Expect = 0.076<br>Identities = 10/13 (77%), Positives = 11/13 (85%), Gaps = 0/13 (0%)<br>> Renamded as plantaricin 2
3	ORF01363	MLKNIKKAFKHNRISDKKLAKVMGG<br>KKSRRCQVYNNGMPTGMYTSC	46	> APD_1473|AP02323|Bactofencin A<br>Length=22<br>Score = 39.7 bits (91), Expect = 4e-007<br>Identities = 15/21 (71%), Positives = 17/21 (81%), Gaps = 0/21 (0%)<br>> Renamded as plantaricin 3
4	ORF01599	LKTTWLASLLVTIFWGAVLGLVVTYL<br>GGAMVEALTATPIVREPFKAMAVGIIL<br>AVMSGLLVTTHH	65	> CAMPSQ3668|Hymenochirin-5B<br>Length=28<br>Score = 20.4 bits (41), Expect = 7.4<br>Identities = 10/25 (40%), Positives = 17/25 (68%), Gaps = 1/25 (4%)<br>> Renamded as plantaricin 4
5	ORF01790	MPESTEEIKKMEALIAKLDEQQKQLK<br>AKKRFLRNRLSQQARKARTKRLIEKA<br>LY	54	> DBAASP_862|970|M-zodatoxin-Lt2a Latarcin-2a Ltc-2a<br>Length=26<br>Score = 21.9 bits (45), Expect = 1.2<br>Identities = 10/21 (48%), Positives = 13/21 (62%), Gaps = 0/21 (0%)<br>> Renamded as plantaricin 5
6	ORF02155	MKSLDKIAGLGIEMAEKDLTTVEG<br>GKNYSKTWWYKSLTLLGKVAEGTS<br>SAWHGLG	55	> Bagel_II_172|179.2|Plantaricin_N<br>Length=55<br>Score = 116 bits (291), Expect = 1e-036<br>Identities = 55/55 (100%), Positives = 55/55 (100%), Gaps = 0/55 (0%)
7	ORF02163	MKKFLVLRDRELNAISGGVFHAYS<br>ARGVRNNYKSAVGPADWVISAVRG<br>FIHG	52	> Bagel_II_169|176.2|Plantaricin_F<br>Length=52<br>Score = 108 bits (270), Expect = 2e-033<br>Identities = 52/52 (100%), Positives = 52/52 (100%), Gaps = 0/52 (0%)
8	ORF02164	MLQFEKLQYSRLPQKKLAKISGGFN<br>RGGYNFGKSVRHVVDAIGSVAGIR<br>GILKSIR	56	> Bagel_II_168|175.2|Plantaricin_E<br>Length=56<br>Score = 112 bits (281), Expect = 5e-035<br>Identities = 56/56 (100%), Positives = 56/56 (100%), Gaps = 0/56 (0%)
9	ORF00645	MTKRQHYRPVYAKTRWARWRYRL<br>GWLLVLLVIIGSVWGGLAWLRWRS<br>DAVVSGFDVRGVAVSQNDGYLDFA<br>ALQNDGLKFVYLHATQGASYTDDN<br>FASNYERIVGTSLGVGVIHTFSFSST<br>AAAQAAYFEKTVGDSIGNLPIAIQV<br>QYYGDYTDQTIAVRKSRAKLKALV<br>TTLTQDYNRSCVVWSTPAVAKQIV<br>KPALKDTDLWLDTAKTHQQGRRV<br>MFMHYSDRAVYRQNGTRQEFAGIL<br>FNGSVTAYNKVVAQGLN	258	> Lysozyme M1<br>Compositional matrix adjust. 257/258(99%) 258/258(100%) 0/258(0%)
10	ORF02421	MKKISFKNADGSLNGKLIAGIISLLI<br>VLIQQIFAMFGIKFTGDWSAIVAVIN<br>TVLTILGMLGVITDVQTVTVPTVKS<br>DEESQVEATANKVADEAQTPTSTV<br>AAVNSSASSNTETTSESASQSGEKV<br>V	127	> Bacteriophage holin<br>Compositional matrix adjust. 127/127(100%) 127/127(100%) 0/127(0%)

Rows 1–5 (yellow): Uncharacterized proteins; rows 6–10 (white): Known proteins.

Table 2. Transcriptomic analysis results of AMPs from *L. plantarum* NIBR97. The AMP transcripts were examined by RNA-sequencing. Lplan-ON and Lplan-OD05 indicate total RNA samples extracted from *L. plantarum* NIBR97 grown during stationary and exponential phases, respectively.

Test_id	SampleA	SampleB	logFC	Absolute Fold Change	logCPM	*p* Value	FDR	Start	End	Strand	Description	Renamed
ORF00467	Lplan-ON	Lplan-ODO5	-0.086799	1.0620114	2.5828759	0.9523457	1	17279	17737	+	hypothetical protein	Plantaricin 1
ORF01336	Lplan-ON	Lplan-ODO5	-0.270876	1.2065403	4.3647043	0.84403	1	30744	30920	+	hypothetical protein	Plantaricin 2
ORF01363	Lplan-ON	Lplan-ODO5	-0.285383	1.2187342	2.2191936	0.8435393	1	2926	3066	-	hypothetical protein	Plantaricin 3
ORF01599	Lplan-ON	Lplan-ODO5	0.1082445	1.0779158	2.5404791	0.9455275	1	105927	106124	-	hypothetical protein	Plantaricin 4
ORF01790	Lplan-ON	Lplan-ODO5	0.0224743	1.0156999	2.5730455	0.9946698	1	1288	1452	+	hypothetical protein	Plantaricin 5
ORF02155	Lplan-ON	Lplan-ODO5	0.9820619	1.9752865	1.2564042	0.4931495	1	96779	96946	+	Plantaricin_N	
ORF02163<br>ORF02164	Lplan-ON	Lplan-ODO5	-0.322824	1.2507762	1.0346486	0.8287364	1	102587	102745	-	Plantaricin_F<br>Plantaricin_E	
ORF00645	Lplan-ON	Lplan-ODO5	-0.560874	1.4751622	5.7206241	0.6808994	1	16330	17106	-	Lysozyme M1 precursor	
ORF02421	Lplan-ON	Lplan-ODO5	-1.349274	2.5478396	3.0879399	0.3336474	1	148606	148989	-	Bacteriophage holin	
